# On the Treatment of Internal Strabismus

**Published:** 1903-07

**Authors:** Dunbar Roy

**Affiliations:** Atlanta, Ga.


ATLANTA

### Journal-Record of Medicine.

**Successor to Atlanta Medical and Surgical Journal, Established 1855, and Southern Medical Record, Established 1870.**

VOL. V. JULY, 1903. No. 4.

BERNARD WOLFF, M.D., EDITOR, Nos. 319–20 Prudential.

Published Monthly.

M. B. HUTCHINS, M.D., BUSINESS MANAGER, No. 64 Marietta St.

### *ORIGINAL COMMUNICATIONS.*

### ON THE TREATMENT OF INTERNAL STRABISMUS.*

By DUNBAR ROY, M.D.,

ATLANTA, GA.

This subject has been suggested to me because its importance does not seem to be fully appreciated by the laity and even by the profession at large. There are two prominent reasons for this condition of affairs.

The first is due to the fact that the itinerant quack, and even some of home production, promulgates the idea that the treatment of strabismus is a very simple thing and can be easily remedied without pain or bandage. The second is due to the laity itself, which seeks to have a defect remedied in the speediest manner possible, many times at the very first examination. It is for the purpose of trying to impress upon the profession at large the importance of a thorough study into the treatment of this condition that the subject is brought before you to-day.

*Read before the Medical Association of Georgia, April, 1903, at Columbus.

Allow me to refresh your memory with a few simple points in physiological optics.

In order for us to have distinct single vision the rays of light from an object must be focused upon corresponding retinal points. This always occurs in so-called normal eyes when the focusing point is on each macula lutea, the point where the focus must fall in order to have the most distinct vision. For us then to have single vision the two eyes must always naturally turn in unison to the same degree.

For instance, the rays coming to the eyes from objects situated at infinity are supposed to be parallel, while those from an object, say one foot, are convergent, and in this latter case both eyes turn in, but the same degree of turning is common to both which brings the rays to corresponding retinal points. Now if rays from an object do not fall upon corresponding retinal points, as when they fall on the macula in one eye and a certain distance from that in the other, then we will have double vision, and their distances apart will depend upon the distance of the separation of the corresponding retinal points. Remembering also the law of optics that an object is always appreciated by the retina as being directly opposite to the point in the retina where the rays are focused; that is, rays from as object below strike the upper part of the retina and the object is therefore recognized as being below. When one eye fixes upon an object and the other eye turns in some other direction, we have the condition known as strabismus.

In the discussion of this subject I shall naturally leave out of consideration the strabismus due to paralysis.

Strabismus is almost universally of two kinds, especially such as is most frequently called upon to be corrected by operative measures.

1. Internal strabismus where the eye turns in. This is capable of subdivision into (a) concomitant, where first one eye will turn in and then the other, or, in other words, where the patient fixes with either eye; (b) periodic, where the squint is sometimes present and sometimes absent; (c) permanent, where the same eye is always squinting.

2. The other most frequent form is external strabismus, where one eye turns out.

These two classes of strabismus, the external and the internal, constitute practically the only classes which we are most often called upon to treat surgically, and of these internal strabismus constitutes almost nine tenths.

The etiology of internal strabismus is still an unsettled question. A number of scientific observers believe that it is due to some anatomical formation of the orbital cavity, on account of which the movements of the eyes are limited and even retarded.

By others the causal factor is placed in some derangement of the nerve centers governing the ocular movements, while others consider the overdevelopment of certain ones of the extrinsic muscles to be the most important factor, and still others believe that it is a lack of development in the fusion center.

That the same etiology cannot be attributed to every case of strabismus is recognized by all careful observers, and for this very reason the same treatment, whether operative or non-operative, is not universally applicable.

I am fully convinced that the proper importance of the treatment of strabismus, or cross-eyes, is by no means appreciated by the laity, and I am even free to admit by the practitioners themselves.

The popular opinion that a case of strabismus can be carried to the oculist's office at any time; that it takes but a moment to cut the muscles and straighten the eyes; that there is no pain whatever connected with the operation, is indeed fallacious.

The sooner the general practitioner and likewise the public are taught that cases of strabismus must be studied scientifically and thoroughly before any operative intervention is undertaken, the better will it be for the patients so afflicted.

It is very easy to straighten an eye by cutting the tendons of the muscles which turn the eye too much in that direction, and while the immediate effect looks brilliant the remote results are frequently unsatisfactory. You must remember that a patient afflicted with strabismus must in time depend upon these ocular members for securing an education and for the earning of his daily bread. For this reason, in correcting such a deformity, the physician must consider the utilitarian feature of his patient's eyes and not the cosmetic result. In other words, it is not *straight* eyes which are of most value to the patient, but *strong* eyes which will allow him to do his daily work, and when the two can be combined we have the ideal result.

My own ideas in regard to the treatment of internal strabismus are based upon personal observation and experience both in my own cases and in those of other oculists, and from a study of the literature which has been written upon this subject by men of wide clinical experience and judgment. I would divide the treatment of internal strabismus into three parts or methods, believing, as I do, that this is the only scientific way of managing these cases.

1. *By Glasses.*—Ever since the days of Donders, the father of scientific ocular refraction, it has been daily noted by oculists that there is a close relationship between strabismus and refractive errors of the eye. The fact has been noted that with internal strabismus there is usually associated hypermetropia or far sight, and with external strabismus myopia or near sight.

It has also been noted that with many cases of internal strabismus associated with hypermetropia the correction of the latter would sometimes cause a disappearance of the former, and for this reason the manner of correcting internal strabismus by the use of glasses is a well-recognized principle of treatment.

The explanation of this is found in the fact that the internal recti and ciliary muscle respond to the same nerve stimulus, and if therefore eyes are afflicted with hypermetropia, a stronger impulse must be given to the ciliary muscle in order to see distinctly than if the eye was normal, and this extra amount of impulse must also stimulate the internal recti to overactivity, which it does not need, but which is manifested by the turning in of one of the eyes.

The younger the patient the more satisfactory and the more certain is the correction of internal strabismus by means of glasses. The reason for this is largely due to the fact that an eye that has had a constant squint for some time becomes amblyopic or partially blind, and if it should not be exercised by having its refractive error corrected and its function brought into use, what little vision that does remain will become deteriorated. Hence the necessity for correction of refractive errors associated with internal strabismus before there is any difference in the vision of either eye.

My practice in every case of internal strabismus is to correct first every particle of refractive error by the prolonged use of atropin and correcting lenses, and to give this a trial for months before undertaking any operative procedures.

I am firmly convinced that the man who does not do this is treating his patient with grave injustice. It does not matter how young the child is, this procedure can be carried out in at least some modified form in every case. It may look cruel to put glasses on a very young child, but is this not better than to have a lasting deformity, with chances of good sight in only one eye and later in life to be operated upon with uncertain results? I have put glasses upon children as young as three years, and, in every case I have been able to correct their internal strabismus. My experience is also in accord with that of other competent observers, in that a full correction must be given if a sure result is desired.

Mothers should be taught by their family physician to note early in life any deviations in the eyes of their children, and to early take the case to a conscientious oculist so that the defect may be remedied if possible while yet there is time.

You may ask how are we to tell in children so young whether the existing internal strabismus is due to a refractive error? No case of refractive error can be properly estimated until the ciliary muscle is paralyzed by means of a mydriatic. If a mild solution of atropin is dropped into the young child's eye, and if when the pupils become widely dilated the deviation disappears, then it is almost self-evident that a refractive error exists.

If the child is in any degree old enough, the amount of error should be corrected with glasses. Fortunately we do not have to depend upon the answers of the patient to tell whether or not we have the correct glass, for by means of retinoscopy or the shadow test the exact refractive error can be estimated and the proper glass prescribed with every degree of certainty. In fact, for the fitting of glasses for any one, all other tests are insignificant as compared with retinoscopy.

During the last year I have corrected an internal strabismus in two cases of children three years old who are wearing glasses without the least bit of trouble.

I am firmly convinced that no child should be operated upon for internal strabismus until a prolonged use of conservative methods has been tried.

2. *Optical.*—By this I mean a systematic use of the eyes singly or combined, with instruments or without, which will exercise the power of vision and also the power of fusion.

It is a recognized fact that the squinting eye of a patient who has suffered with internal strabismus for some time is almost always amblyopic or defective in vision.

This I believe to be due in the first place to inability to fuse the two images, and as a result of this the loss of exercise to the visual function. In an old case of strabismus the patient is not annoyed by double vision, whereas in one of short duration this is a symptom which is frequently present. The cause of this is the fact that in cases of long standing the patient soon learns to do all his work with one eye and ignores the image produced in the squinting eye. It is only after long practice that a patient can do this.

Now if a case which can be corrected with glasses be not corrected early in the disease, then it is hard to overcome this defective vision in the squinting eye.

Hence, even when the refraction has been corrected, both before and even after an operation, the amblyopic eye must be exercised by placing a pad over the best eye and thus make the patient use the defective member. Another method is to put atropin in the good eye and thus necessitate the use of the bad one. This is on the principle that any portion of our body will grow weak unless exercised. It is sometimes remarkable how vision will improve in a defective eye when such exercise is given.

In conjunction with the above the fusion power of the two eyes must be brought into play, and this is best done by the use of the stereoscope. Even the ordinary instrument can be used with success. A better apparatus and one with which I am much pleased, is the Worth amblyoscope. This is an instrument on the principle of the stereoscope, and with children it can be used with advantage because it always entertains as well as exercises. It works on the principle of fusing two half objects into one.

Careful and conscientious oculists have in the last few years depended much upon this optical treatment of internal strabismus, since it can be readily shown to be the rational and scientific treatment in a great many of these cases.

Dr. Gelpe, of Carlsruhe, in an extended article published last year upon the treatment of internal strabismus, sums up his views in the management of these cases in a manner which is probably in accord with the views of most of the ophthalmologists of the present day. His method of dealing with these cases varies according to the age of the individual.

Up to six years of age the eyes of the patient are treated prophylactically, that is, the general condition of the patient is watched and the use of the eyes more especially.

From the sixth to the twelfth year, optical treatment is instituted, that is, by means of glasses, stereoscopic exercises, atropia, etc. After this operative treatment is used if the previous treatment has failed, and even this is followed by careful testings, exercises, etc., in order to produce permanent binocular vision as far as possible.

*Operative.*—If after such careful treatment as has been outlined improvement does not follow, then of course we must have recourse to operative procedures. Here also ophthalmologists differ as to the best operation. I believe it is not saying too much in stating the fact that the majority of oculists of to-day believe that the advancement operation, or advancement with cutting of the opposing muscle, is much to be preferred to the old operation of cutting freely both the interni muscles.

Time does not permit me to go into details of the operative technique of advancement, nor would it be of any great benefit to my hearers, since such must vary according to the individual case and to the preference of each operator.

The quick cutting of both interni muscles and the immediate straightening of the eyes may indeed appear brilliant, as I have previously stated, but if the history of these cases could be followed for years we would often find that the remote results are far from satisfactory. Such remote results as turning out of the eyes, sinking in of the caruncles, inability to use the eyes at close work because of the weakness of the interni muscles, are conditions which cannot be overlooked. In mild cases of concomitant periodic squint, where optical treatment has not afforded a good result, the partial cutting of both interni muscles will frequently prove entirely satisfactory.

When, however, the squint is permanent in one eye and of high degree, the advancement of the externus and tenotomy of the internus of the same eye have in my experience proved to be the best operation. When the squint is concomitant and of high degree, the advancement of both external recti is an operation which is rapidly gaining in favor.

In conclusion, allow me to say that my main object in presenting this paper was to urge upon physicians the recognition of its importance and the importance of treating each case in a thoroughly scientific manner. Operation should never be done until all other measures have failed after prolonged trial. Whatever operative procedure is adopted, such should be done with the same care and precaution as any other major surgical operation, and the laity should be made to realize this importance if the best results are to be obtained.

*Grand Opera House.*

